# A bibliometric and content analysis of strategy-based instruction and self-regulated learning in second or foreign language teaching from 1994 to 2024

**DOI:** 10.3389/fpsyg.2025.1474689

**Published:** 2025-01-29

**Authors:** Ruihua Chen, Xiao Wang, Kezhen Zhu

**Affiliations:** ^1^Sichuan Normal University, Chengdu, Sichuan Province, China; ^2^Business School, Chongqing City Management College, Chongqing, China

**Keywords:** strategy-based instruction, self-regulated learning, English as second or foreign language teaching, bibliometric analysis, systematic review, content analysis

## Abstract

**Introduction:**

This study aims to explore the research themes and identify gaps on strategy-based instruction (SBI) and self-regulated learning (SRL) in language education, spanning the period from 1994 to 2024. Using a systematic review methodology, this study examines the empirical research in the SBI and SRL in English as second or foreign language teaching contexts published during this period.

**Methods:**

The review employs both bibliometric and content analysis to reveal current trends to facilitate understanding of the role of SBI and SRL in teaching practices. Using VOSviewer, a keyword co-occurrence analysis was conducted on 35 selected papers from Web of Science and Scopus databases.

**Results:**

The bibliometric analysis reveals a shift in research focus from student self-development to the impact of learning strategies on students’ learning achievement. The content analysis indicates that studies have focused on writing instruction, enhancement of learners’ SRL, and technology-assisted instruction, with the latter showing potential for developing students’ SRL strategies.

**Discussion:**

Overall, this study highlights the significance of integrating SBI and SRL into language education, offering insights into writing instruction, technology-assisted learning, and SRL development. It emphasizes the need for future research to explore specific skills, technology-assisted learning, longitudinal mixed-methods approach. This paper aims to serve as a useful resource for researchers and practitioners involved in research related to EFL and ESL teaching.

## 1 Introduction

The role of strategy-based instruction (SBI) and self-regulated learning (SRL) in English as a foreign language (EFL) and English as a second language (ESL) contexts has received increasing attention in recent years. These strategies have shown promise in enhancing language acquisition. For instance, recent studies have examined how integrating SRL strategies into writing instruction can support students with writing difficulties (Bewley, 2020; Smith et al., 2020). Similarly, a few studies have examined the role of reading strategies and their influence on enhancing learning outcomes (Li et al., 2022; Li and Gan, 2022; Li et al., 2024). Studies in other language domains, such as speaking (Uztosun, 2020), vocabulary (Araya Pérez et al., 2013; Fatemipour and Najafgholikhan, 2015; An et al., 2021), writing (Woo and Kim, 2024) and grammar (Alkhalaf, 2023) have also reported positive effects of SBI and SRL on learning performance. However, the evidence is not universally conclusive. For example, some studies found no significant impact of strategy-based instruction on students’ self-regulation abilities (Hsu et al., 2024), strategy use, motivation, and self-efficacy (Li et al., 2022; Chen et al., 2021), or overall SRL (Meşe and Mede, 2023). Additionally, existing studies often focus on isolated language skills or specific learner groups, limiting the understanding of the broader applications of SBI and SRL. These inconsistencies suggest the need for a systematic evaluation of SBI and SRL, addressing how these approaches function in diverse educational scenarios and which factors influence their effectiveness.

This study aims to address the gap in systematic evaluations of SRL and SBI in EFL and ESL contexts by exploring key research themes and examining their roles in teaching and learning. Using bibliometric and content analysis of data from both Web of Science and Scopus database, the research provides valuable insights into current research focuses, highlights underexplored aspects, and identifies future directions, contributing to a deeper understanding of how SRL and SBI can be effectively implemented and studied in language education.

## 2 Literature review

### 2.1 Self-regulated learning strategies

Research in the domain of psychology have long investigated the capability of individuals to regulate, adapt, and manage their own cognitive processes. The concept of SRL was initially proposed by Zimmerman (1986), defining it as the active involvement of learners in metacognitive, motivational, and behavioral aspects during the learning process. Early studies focused on distinguishing SRL from metacognition and investigating the significance of SRL within the learning process (Zimmerman, 1986; Pintrich et al., 1993). Later, SRL was conceptualized as learners’ systematic management and regulation of their learning process to achieve learning goals (Pintrich, 2004; Zimmerman and Schunk, 2011). More recently, according to Zimmerman (2011), SRL refers to “the ways that learners systematically activate and sustain their cognitions, motivations, behaviors, and affects, toward the attainment of their goals.”

As SRL evolves into a key conceptual framework within the field of education, numerous models have emerged to explain and predict learning behaviors. Zimmerman’s (2000) model of SRL highlights the critical role of goal-setting in the learning process. Clear and specific learning objectives enable learners to predict behaviors most conducive to achieving their outcomes (Zimmerman, 2000). In Pintrich’s (2000) model, the monitoring phase involves learners assessing their cognitive, motivational, and behavioral states. This phase includes engaging in cognitive monitoring activities, such as making judgments about their learning progress (Pintrich, 2004). Influential models by Boekaerts and Niemivirta (2000), Pintrich (2000), Winnie and Hadwin (1998), and Zimmerman (2000) examine various aspects of SRL, such as cognition, motivation, and affective, deepening the understanding of the learning process. A key feature in these models is the integration of self-efficacy, as seen in the work of Pintrich (2000) and Zimmerman (2000). Self-efficacy refers to learners’ evaluations of their capabilities to achieve specific levels of performance (Schunk et al., 2014). It plays a crucial role in research on SRL, influencing learners’ motivation, behavior, and achievement-building blocks of SRL. Pintrich (2000) pointed out that learners’ learning objectives shape how learners manage their learning processes and their levels of confidence in their abilities. The relationship between learning objectives and learners’ level of confidence highlights how specific leaning objectives can strengthen learners’ belief in their capacity to accomplish tasks. Zimmerman (2000) further describe self-efficacy as a primary driver for learning motivation and achievement. Besides, the Strategic, Self-Regulation (S^2^R) Model proposed by Oxford (2016) offers additional insights into SRL processes. The S^2^R model categorizes strategies into cognitive, affective, sociocultural-interactional, and metastrategies (Oxford, 2016). The author considers “metastrategies” as a comprehensive category, because they contribute to the control of cognitive, affective, and sociocultural-interactional language learning strategies. Essentially, metastrategies interact with and influence the other three types of strategies, making them integral to the SRL framework.

Despite the various approaches to SRL, it is generally agreed that SRL is a complex construct encompassing multiple facets, including cognitive, metacognitive, behavioral, and self-motivational aspects (Zimmerman and Schunk, 2011, 2012). Cognitive strategies refer to learners’ ability to process information when completing tasks (Pintrich, 1991). Metacognitive strategies are described as learners’ capacity to monitor and manage cognitive processes, including goal setting, learning planning, monitoring learning processes, and evaluating learning achievements (Winne, 2011). Metacognitive strategies provide learners with internal assessment when facing learning tasks. Social-behavioral strategies refer to learners’ regulation of learning behaviors in specific contexts (Zimmerman, 2011). Finally, motivational strategies refer to the deliberate procedures employed by students to maintain or enhance their engagement and interest in learning tasks (Wolters, 1999).

Recent research has explored interventions to improve SRL among English language learners. In a study by Karami et al. (2019), SRL strategies can be improved through electronic portfolio, which refers to the use of digital platforms to record and showcase students’ work. This allows both students and teachers to engage in self- and peer assessment, therefore facilitating the development of students’ SRL (Kumar et al., 2023). Additionally, Seker (2016) found that scenario-based SRL instruction significantly enhance the use of affective and social strategies, but its impact on cognitive and metacognitive strategies seems limited. However, longer durations of instruction may have a notable influence on these areas (Seker, 2016). Similarly, the study by Guo et al. (2021) examined the effects of process-based instruction (PBI) on the application of SRL in EFL writing and noted varied levels of SRL utilization across writing stages, with the pre-writing stage being particularly influential for fostering SRL. Another line of research exploring self-regulated strategy development (SRSD) has demonstrated its effectiveness in improving academic performance and SRL skills (Alreshoud and Abdelhalim, 2022). It was discussed that the SRSD framework helps to facilitate students’ autonomous application of strategies and self-regulation techniques, such as setting objectives, monitoring, self-evaluating, and self-reinforcement (Harris et al., 2008). Graham et al. (2005) proposed five steps of SRSD, including discuss it, model it, memorize it, support it, and independent performance. Chen et al. (2021) latter added a preliminary step, which is to develop students’ background knowledge. This addition ensures learners are adequately prepared for subsequent steps. The SRSD model is widely employed in writing instructions. For example, De La Paz and Sherman (2013) applied SRSD to the revision stage of writing instruction and found that students who received SRSD instruction made more meaningful revisions. Additionally, Alreshoud and Abdelhalim (2022) demonstrated that SRSD has a significantly positive impact on students’ reading skills and reading self-efficacy.

Also, researchers explored the use of SRL questionnaires to acquire a deeper understanding of students’ utilization of SRL strategies across various learning contexts. The Metacognitive Awareness Inventory (MAI), developed by Schraw and Dennison (1994), is designed to assess students’ levels of metacognitive awareness, which refers to learners’ cognitive and understanding of their own learning processes. Another widely used SRL scale is the Motivated Strategies for Learning Questionnaire (MSLQ) (Pintrich, 1991). The MSLQ seeks to evaluate students’ learning behaviors by assessing aspects such as learning strategies, motivation, and learning environment. Weinstein et al. (1987) developed Learning and Study Strategies Inventory (LASSI), which employs the self-report to assess learners’ understanding and implementation of learning strategies. Grounded in self-regulation theory (Zimmerman, 2011), Teng and Zhang (2016) developed the Writing Strategies for Self-regulated Learning Questionnaire (WSSRLQ). This questionnaire measures the use of cognitive, metacognitive, and social behavior strategies by English language learners, enabling educators to design interventions that enhance their learning capabilities and academic performance (Moseki and Schulze, 2010). Uztosun’s (2020) research expanded this approach by designing a scale to assess learners’ self-regulated motivational orientations, focusing on improving speaking skills. These questionnaires provide quantitative insights into students’ SRL strategies, allowing researchers to pinpoint areas for improvement.

The application of SRL strategies to specific language skills, particularly writing, has also been extensively studied. A range of studies discovered that integrating instruction on SRL with writing strategies could significantly enhance students’ writing proficiency (Glaser and Brunstein, 2007; Teng and Huang, 2019; Woo and Kim, 2024). Of note, Tian et al. (2022) investigated the role of SRL strategies in feedback revision processes for L2 writing, finding that motivational strategies were the most employed among cognitive strategies, metacognitive strategies, social strategies, and motivational strategies. While these studies support the critical role of SRL in enhancing writing proficiency among English language learners, a few studies present contrasting findings. Chen et al. (2021) reported that self-regulated strategy development (SRSD) instruction during the text revision phase in writing has no impact on students’ self-efficacy. Teng and Zhang (2020) found that SRL-based writing instruction improves short-term writing performance but has limited long-term effects. Beyond writing, SRL strategies has been documented to play a positive role on speaking proficiency (Wang and Sun, 2024) and vocabulary acquisition (Fatemipour and Najafgholikhan, 2015).

Recent studies have begun to focus on technology-based SRL strategies, which use tools such as online electronic dictionaries, translation software, and digital media to enhance their English language skills and learning performance (An et al., 2020, 2021; Steel and Levy, 2013; Lai et al., 2018). Specifically, An et al. (2021) suggested that technology based SRL strategies can enhance the self-efficacy of EFL learners. Similarly, Chang and Chang (2014) investigated Taiwanese university students’ listening comprehension strategies by employing YouTube and found that metacognitive instruction significantly enhanced students’ listening comprehension abilities. Overall, the majority of findings highlight the effectiveness of SRL strategies in enhancing English language learning and improving student performance. However, a few studies present contrasting findings, emphasizing the need for further exploration into the conditions under which SRL is most effective.

### 2.2 Strategy-based instruction

Strategy-based instruction (SBI) refers to an approach that aims to improve the utilization of strategies during the learning process by incorporating strategy training into a standard language learning curriculum (Rubin et al., 2007). Studies suggested that effective language strategy-based instruction can lead to successful language learning, such as language skills (Chamot and O’Malley, 1994) and higher retention rate of new words (Cohen, 2014). Currently, there is a growing trend of exploring the integration of language learning strategies into foreign language classrooms to enhance learning outcomes (Chamot, 2005). This has led to the emergence and increasing adoption of strategy-based instruction. The purpose of SBI is to promote the development of learner self-management skills, because it can be difficult for learners to choose appropriate learning strategies when they do not have specific tasks or goals. Several instruction models have been devised for training language learning strategies in second language settings. Rubin et al. (2007) identified four steps as the core features of an SBI model: (1) raising awareness of existing strategies, (2) teacher presentation and modeling, (3) providing multiple practice opportunities, and (4) self-evaluation and strategy transfer. A well-defined SBI model that incorporates all the above four features is the Cognitive Academic Language Learning Approach (CALLA) by Chamot and O’Malley (1994). This model fosters learners’ cognitive and language development through integrating language, and strategy training. Another popular taxonomy of language learning strategies in general language teaching research is proposed by Oxford (1990). The author categorizes strategies into six parts, namely memory, cognitive, compensation, metacognitive, affective, and social strategies.

The effectiveness of Strategy-Based Instruction (SBI) in improving specific language skills among learners has been well documented. For instance, Thompson and Rubin (1996) used authentic video material in a real classroom to assess the effectiveness of teaching cognitive and metacognitive strategies in enhancing listening comprehension. Similarly, listening instruction based on metacognitive strategies can help students become more concentrated and plays a significant role in the long-term development of listening proficiency (Siegel, 2014). Further, differentiated instructions are observed to have a positive impact on students’ oral reading fluency and comprehension abilities (Reis et al., 2011). Nevertheless, empirical evidence regarding the efficacy of SBI in English language acquisition remains inconclusive. Li et al. (2022) reported no significant impact of CALLA-based reading strategy instruction on students’ strategy use, motivation, and self-efficacy. Similarly, Meşe and Mede’s (2023) research found that while the implementation of differentiated instruction improve English speaking proficiency, it had little effect on overall SRL. Guo et al. (2021) examined the impact of process-based instruction (PBI) on Hong Kong students’ deployment of SRL strategies in writing tasks. The findings indicate that the frequent implementation of PBI does not necessarily lead to a higher frequency of SRL strategy use among students. Beyond its direct effects on language learning outcomes, researchers have explored the contextual elements that influence the success of these instructions. While numerous studies have confirmed the positive impact of SBI on language learning, Carrell (1998) proposed that successful strategy instruction depends on instructional context, including learners’ proficiency levels. For example, studies by Green and Oxford (1995) and Wharton (2000) employed the Strategy Inventory for Language Learning (SILL) to analyze learners’ language learning strategies, and the results demonstrated a positive relationship between learners’ proficiency levels and the use of strategies. However, Hong-Nam and Leavell (2006) suggested that this relationship is not linear. Compared to students with beginning and advanced proficiency levels, those with intermediate proficiency levels showed the most prominent ability in utilizing learning strategies (Hong-Nam and Leavell, 2006). Besides, research also considered the roles of learner motivation, cognitive styles, and language aptitude, in influencing the effects of SBI on language learning (Ehrman and Oxford, 1995; Skehan, 1998).

Overall, the study of SBI in EFL contexts has contributed significantly to the understanding of effective language teaching practices. Through investigating the role of SBI on language learning outcomes and exploring innovative instructions, researchers can help to enhance the effectiveness of SBI for the English language learners.

### 2.3 SRL and SBI in English language learning contexts

SRL and SBI are two significant components in English language learning contexts, and understanding their roles is essential for educators aiming to optimize teaching strategies. A stream of studies have explored the impact of implementing SBI on students’ use of SRL strategies and learning performance. For instance, Kavani and Amjadiparvar (2018) conducted an intervention study, in which the experimental group received instruction centered on six reading strategies, including making connections, predicting, questioning, monitoring, visualizing, and summarizing. Their findings suggest that teachers should consider incorporating SBI into teaching practices to foster more effective language learning. Through meta-analysis, Ardasheva et al. (2017) demonstrated the feasibility of SBI in that employing process-oriented feedback can enhance metacognitive strategies of learners. Instruction based on self-assessment and peer assessment strategies is also one of the effective methods for encouraging students to monitor their learning processes and enhance their’ SRL (Kumar et al., 2023). By prompting students to contemplate, analyze, evaluate, and resolve issues in their learning process, these strategies also foster critical thinking (Zimmerman, 2000). Additionally, Ashraf and Mahdinezhad (2015) found a positive influence of peer-assessment on EFL learners’ autonomy, further supporting its value in language education.

In parallel, artificial intelligence (AI), as an information technology program, has been widely applied in language learning, offering benefits such as personalized learning environments, immediate assessment, and increased student engagement (Haristiani, 2019; Huang et al., 2023; Wei, 2023). For example, Kim (2019) found that students who received AI-assisted instruction performed better in grammar learning compared to those who followed traditional teaching methods, while Xu et al. (2023) found that interactive and speech recognition features improve speaking performance and engagement. Ebadi and Amini (2024) also confirmed the significant impact of AI-assisted language learning on the engagement of Iranian EFL learners. However, not all studies report consistent outcomes. For example, AI-assisted vocabulary instruction had a limited impact improving students’ self-regulation abilities (Hsu et al., 2024).

Several studies also assessed the influence of instruction based on SRL strategies in English language learning contexts. Ching (2002) focused on strategy instruction and self-regulated learning instruction for engineering students in ESL contexts. By employing strategies such as planning, revision, peer assessment, and reflection, Ching (2002) demonstrated that incorporating self-regulated learning strategies into strategy instruction can enhance students’ learning performance. The self-regulated strategy development (SRSD) also has a positive impact on students’ learning performance. Leins et al. (2017) demonstrated that self-regulated strategy development based instruction positively impacts the writing abilities of students with learning disabilities. Teng (2022) study also focused on students’ writing performance, indicating that students who received SRL strategy instruction demonstrated improvements in the content, structure, vocabulary, and language of writing. Additionally, students’ self-efficacy and control of learning beliefs were improved.

Although most empirical studies have demonstrated the positive impact of incorporating SRL and SBI in English language learning contexts, there are some issues that need to be addressed, the following research questions are raised:

- What are the focuses on SBI and SRL research in EFL/ESL contexts?
- What is the role of SBI and SRL in EFL/ESL contexts?
- What are the future research directions on SBI and SRL in EFL/ESL contexts?

## 3 Method

### 3.1 Public selection

**Figure 1:**
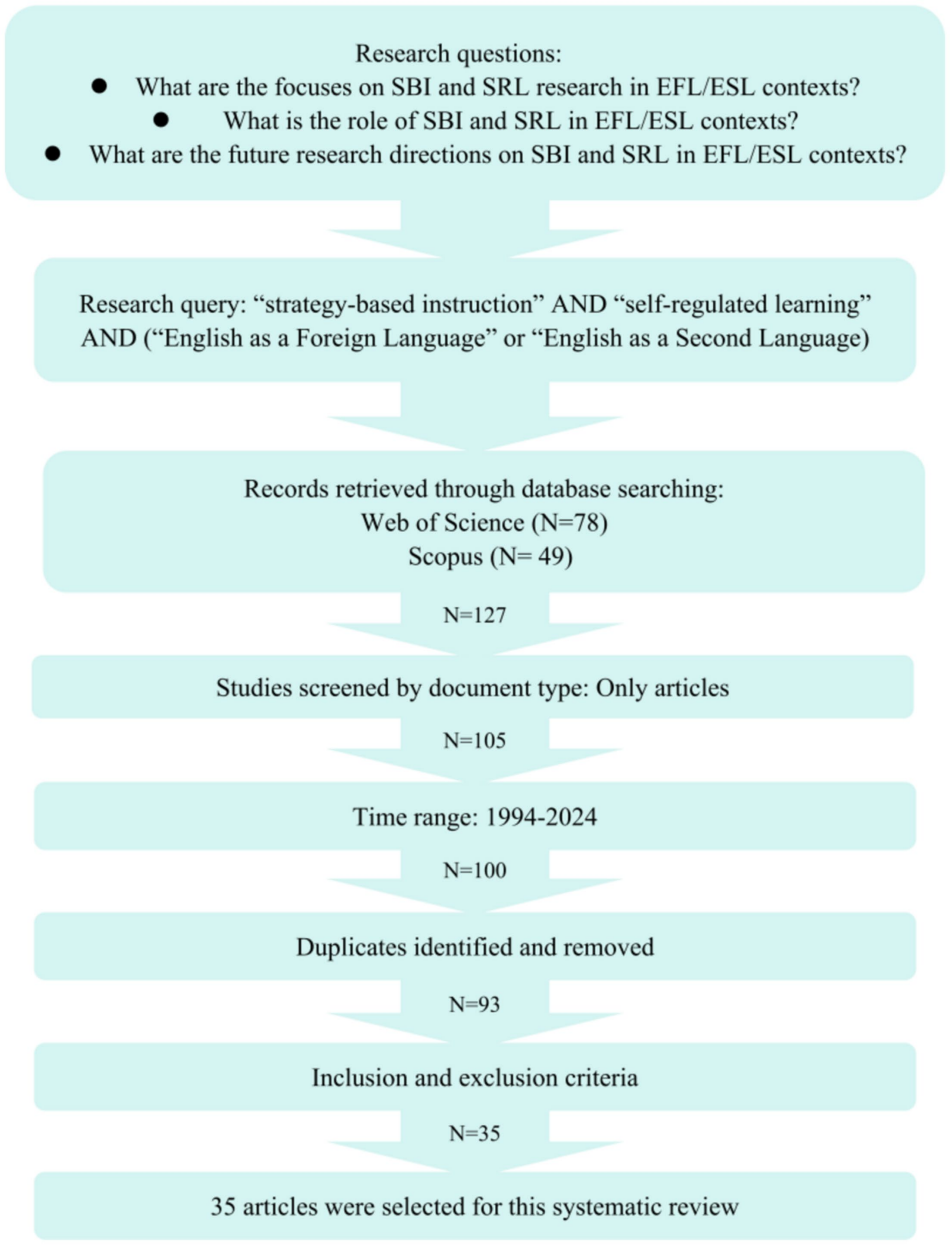
Publication selection procedure.

**Figure 2:**
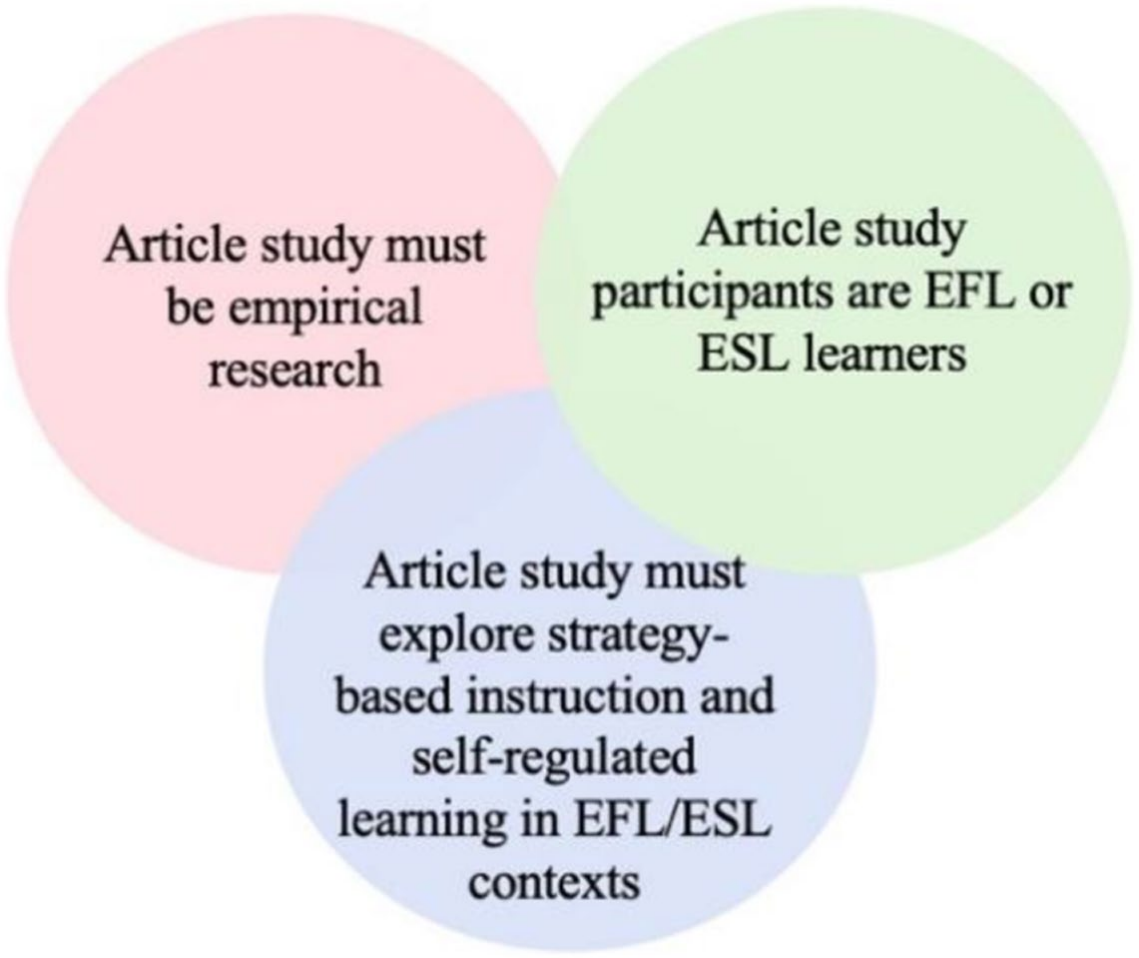
Inclusion and exclusion criteria.

The public selection process was guided by the following procedure to ensure the representativeness and reliability of the selected publications. Firstly, this systematic review collected data from major academic databases such as Web of Science (WoS) and Scopus. WoS, and Scopus are considered the primary databases for systematic review (Singh et al., 2021). Search controlled terms included “strategy-based instruction,” “self-regulated learning,” and “language learning.” The systematic review was restricted to the time frame from 1994 to 2024 to include enough studies, with the language limited to English. A systematic review will be constrained by an insufficient number of relevant studies if the time range is too narrow (Meline, 2006). Thus, the selected time range for this study included all accessible articles. The procedures of this study were constructed relying on the 27-item checklist from Preferred Reporting Items for Systematic Reviews and Meta-Analyses (PRISMA) (Page et al., 2021). After an initial assessment, the abstracts and keywords of each article were further examined to confirm they were consistent with the research aim. The publication selection procedure is presented in Figure 1 and the inclusion and exclusion criteria are in Figure 2.

### 3.2 Bibliometric analysis

Bibliometric analysis is a scientific research method that evaluates the performance of various scientific elements, such as papers, authors, keywords, journals, institutions, and countries, and employs visualization techniques to present the analytical results, thereby measuring scientific output (Donthu et al., 2021; Gutiérrez-Salcedo et al., 2008). Due to its comprehensive and systematic approach, bibliometric analysis has emerged as an increasingly significant method in academic research in recent years (Öztürk et al., 2024). This study employs methods such as keyword co-occurrence analysis and citation analysis to gain an in-depth understanding of strategy-based instruction and self-regulation research. For comprehensive analysis, eligible data were selected from WoS and Scopus, and VOSviewer was used for data analysis. VOSviewer can be utilized to construct co-occurrence networks of literature, analyze the co-occurrence relationships among key terms, assess the interrelations among publications, and aid in identifying research focuses and research trends in the domains of strategy-based instruction and self-regulated learning (van Eck and Waltman, 2010).

### 3.3 Content analysis

Content analysis is a systematic research method designed to analyze and interpret textual or other meaningful data to derive replicable and valid conclusions (Krippendorff, 2018). The study employed content analysis to examine the research objects and learning contexts of the selected studies. Content analysis is conducted using NVivo 14, which is a specialized software application for qualitative data analysis, capable of classifying, coding, and analyzing data (Jackson and Bazeley, 2019). The initial set of items in the coding scheme was created automatically using the NVivo 14 software. Then three researchers independently reviewed and refined the scheme to ensure its quality and accuracy. The inter-rater reliability of three researchers’ coding over 28 items was 0.82.

The investigation was classified into two primary categories: learning context and research objects. The learning context section covered both educational levels and context variety (see Table 1). Teachers and students from primary, secondary, high, and tertiary schools were part of the educational level. The participants of one study included both teachers and students. The studies were conducted in a diverse range of contexts, including mainland China, Iran, Hong Kong, Turkey, Saudi Arabia, USA, Chile, South Korea, Hungary, Thailand, Vietnam, Taiwan.

**Table 1:** Coding scheme for the use of SBI in EFL learning.

Coding items
Learning context
Participants
Teachers
Primary school students
Middle school students
High school students
Tertiary school students
Contexts diversity
Mainland China
Iran
Hong Kong
Turkey
Saudi Arabia
USA
Chile
South Korea
Hungary
Thailand
Vietnam
Taiwan
Research objects
SRL as a strategy instruction
Writing strategies
Speaking strategies
Reading strategies
Self-Regulated Learning Questionnaire
Self-efficacy
Cognitive and metacognitive strategies
The impact of SBI intervention

The section on research objects was categorized into eight coding items, which included SRL as a strategy instruction, writing strategies, speaking strategies, reading strategies, self-regulated learning questionnaire, self-efficacy, cognitive and metacognitive strategies, the impact of SBI intervention.

## 4 Results

### 4.1 Bibliometric analysis findings

#### 4.1.1 Network and overlay visualization of author keywords

**Figure 3:**
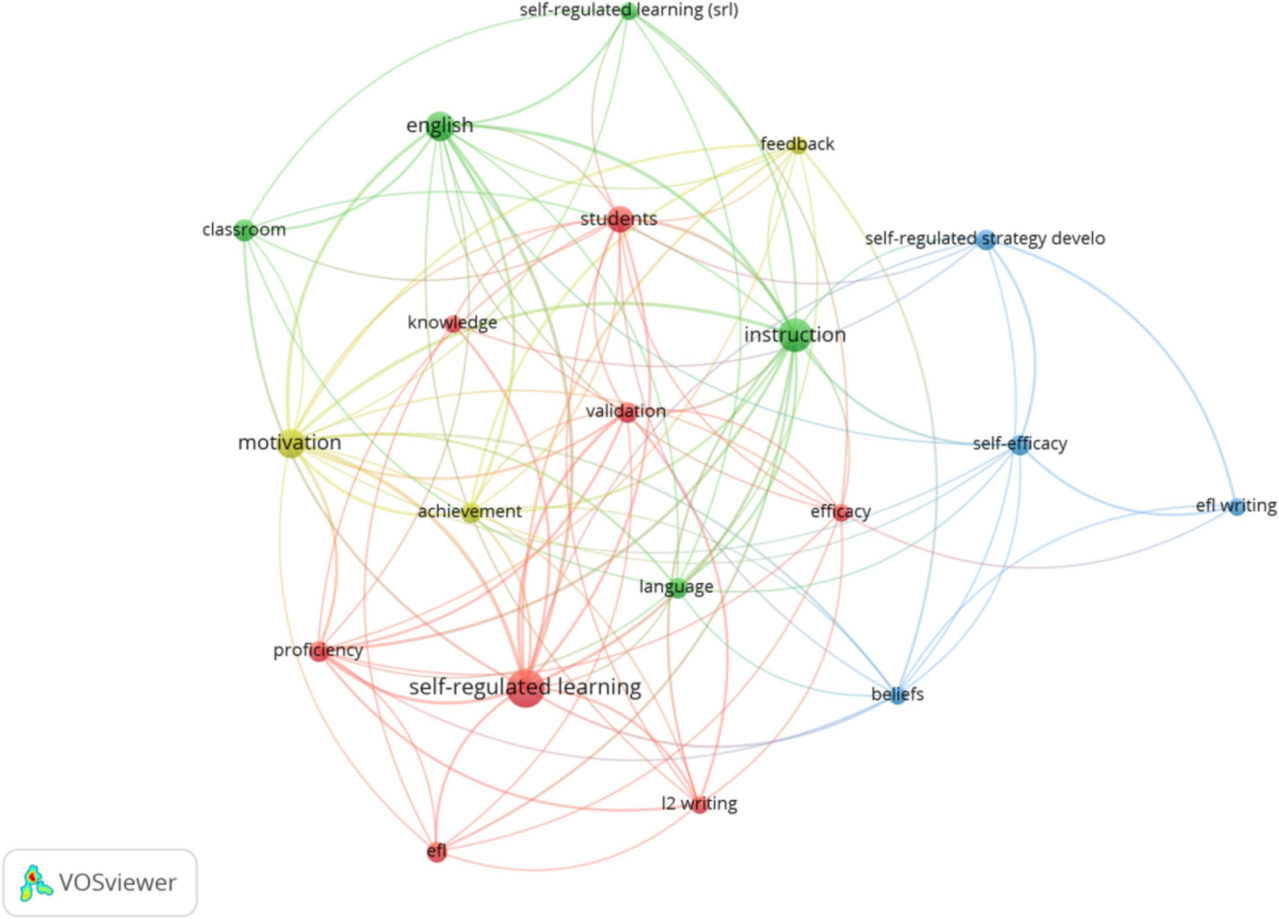
Network visualization of author keywords.

**Figure 4:**
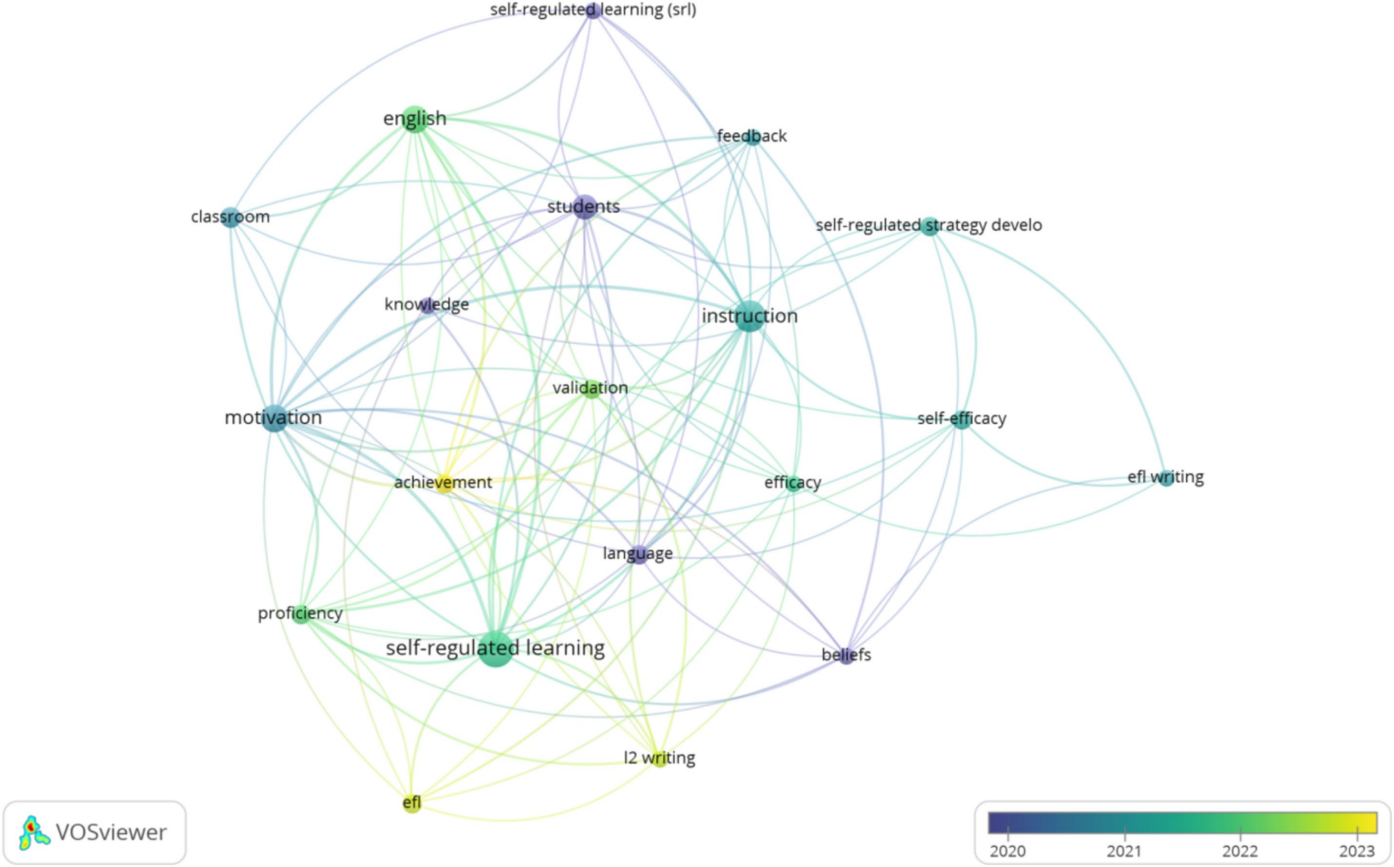
Overlay visualization of author keywords.

Figures 3, 4 demonstrate the network and overlay visualizations of author keywords. The program selects 20 keywords based on the minimum occurrences threshold of 3. The most used author keywords are instruction (*f* = 11), motivation (*f* = 8), English (*f* = 8), students (*f* = 7), and classroom (*f* = 5). Since “self-regulated learning” is a keyword of this study, it was not included in the most used author keywords.

Based on Figure 4, the latest research interests in strategy-based instruction and self-regulated learning areas focus on “EFL,” “achievement,” and “L2 writing.”

The visualizations regarding frequency and trend suggest that most studies have started to focus on students’ overall performance. The focus of research has shifted from student self-development (such as the keywords “students”) to the impact of strategies on students’ learning performance (such as the keywords “achievement”).

#### 4.1.2 Most cited journals in citation analysis

Based on citation analysis, the most cited journals include (Table 2) Modern Language Journal (*f* = 140), Metacognition and Learning (*f* = 112), Journal of Second Language Writing (*f* = 97), Frontiers in Psychology (*f* = 60), and English Teaching-Practice and Critique (*f* = 20).

**Table 2:** Five most cited journals.

Journal	Citations
Modern Language Journal	140
Metacognition and Learning	112
Journal of Second Language Writing	97
Frontiers in Psychology	60
English Teaching-Practice and Critique	20

#### 4.1.3 Major regions publishing studies

Citation analysis was utilized to examine the citation and publication characteristics in relation to regions. The top five regions ranked by publication numbers are presented in Table 3. The region with the most publication numbers is mainland China (publication number = 13). Following are Iran (publication number = 5) and the Hong Kong (publication number = 3). From the table, it can be observed that out of the five main regions, four regions (Mainland China, Iran, Hong Kong and Turkey) are from foreign language teaching and learning contexts, while the remaining region (USA) is from second language teaching and learning contexts. Although the educational backgrounds are distinguished, the theoretical frameworks and designs in different contexts appear to be similar. For instance, some study adopted an experimental group and control group design (Kumar et al., 2023; Sardegna, 2022; Zaien, 2021; Wei, 2023). A few studies have focused on embedding SRL strategies (e.g., planning, monitoring, and evaluation) into technology impacts students’ learning outcomes (Wei, 2023; Regan et al., 2018). However, considering the differences in language use opportunities, language learning goals, and students’ backgrounds, further research is needed to determine whether studies conducted in these two contexts can be verified interchangeably.

**Table 3:** Top five regions ranked by citation number.

Region	Publication number
Mainland China	13
Iran	5
Hong Kong	3
Turkey	3
USA	2

### 4.2 Content analysis findings

#### 4.2.1 The learning context

**Figure 5:**
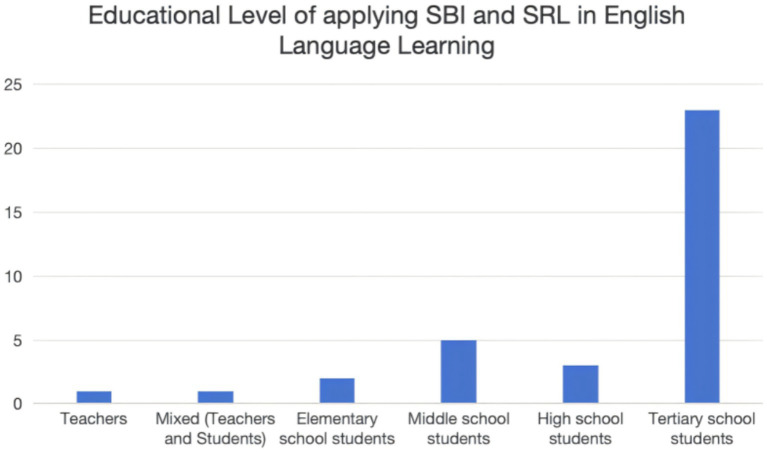
Educational levels of applying SBI and SRL.

The educational backgrounds of participants across these 35 studies are shown in Figure 5. The target language in all 35 studies is English. Therefore, the findings of the following studies are in the context of English as a second language (EFL) and English as a second language (ESL). The results indicate that studies on SRL and SBI in English language contexts have been explored in higher education level, followed by high and middle education levels, and the least in primary education level. Tertiary education, middle school education, high school education, and elementary education level accounted for 66% (23 studies), 14% (five studies), 8% (three studies), and 6% (two studies) of the total number of studies, respectively. At the tertiary education level, one study’s participants were pre-service teachers enrolled in university. One of the studies involved a mixed population of teachers and students who were from the higher education level. 34% (12 studies) of the studies described the students’ English proficiency level, 11 studies are the intermediate level students, and one studies chose students at both intermediate and advanced levels.

**Figure 6:**
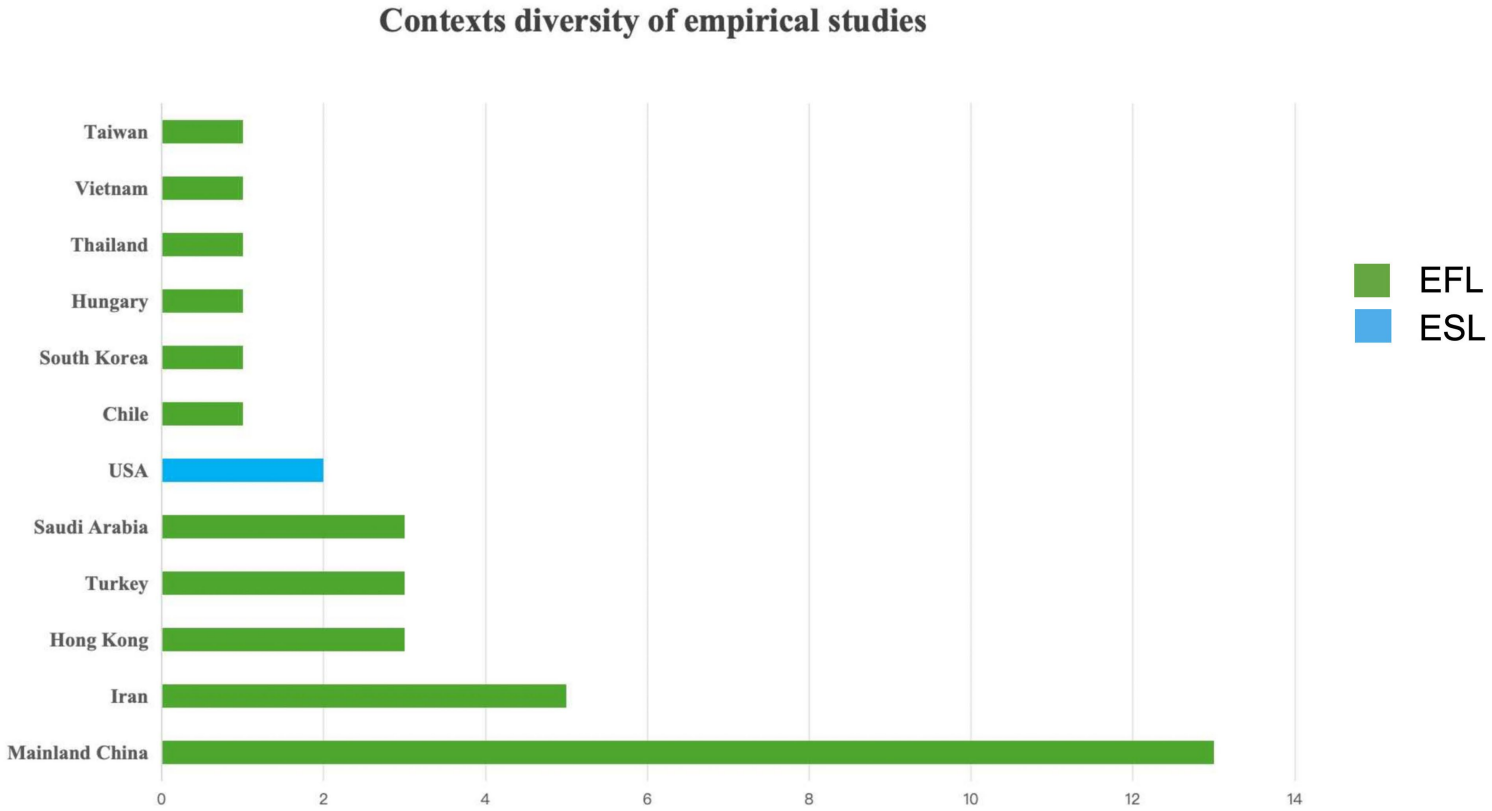
Contexts diversity of empirical studies.

The distribution of research locations on SRL and SBI in the English language learning contexts is illustrated in Figure 6. A total of 10 regions conducted research, mainland China was the highest at 37% (13 studies) of the total studies. Three studies were investigated in Hong Kong, and one study was conducted in Taiwan. Next is Iran, accounting for 14% (five studies), followed by the United States at 6% (two studies). Researchers from Saudi Arabia and Turkey have each conducted three studies focusing on the uses of SRL and SBI in the context of EFL, accounting for 5% of the total studies, while the United States contributed 6% with two studies. Chile, South Korea, Hungary, Thailand, and Vietnam conducted one study exploring SRL and SBI in the EFL context, respectively. In particular, the two studies conducted in the United States were from the theoretical perspectives of ESL approaches, and the subjects of both studies were international students from various regions. Most studies were conducted from the theoretical perspective of the SRL. Three studies were conducted based on social cognitive theory (Zimmerman, 2000). The Strategic, Self-Regulation (S2R) Model (Oxford, 2016) was employed in two studies.

#### 4.2.2 Research objects

46% (16 studies) explored the role of SRL as a strategy instruction in EFL contexts. Among these 16 studies, 50% (eight studies) focused on the impact on students’ writing skills, while 13% (two studies) examined reading and 13% (two studies) explored speaking, and two studies focused on grammar and vocabulary, respectively. Two studies focused on the questionnaire. One study validated The Writing Strategies for Self-Regulated Learning Questionnaire at the tertiary educational level. Another study employed a questionnaire to assess the frequency of students’ use of self-regulated learning strategies in writing. It is worth to note that one study focused on students with learning disabilities in the investigation of writing. In addition, close to 9% of cases (three studies) analyzed reading comprehension abilities, with one case especially addressing the relationship between grammar, vocabulary, and reading comprehension.

Approximately 20% (seven studies) investigated the impact of technology-assisted instruction on students’ learning performance and SRL strategies. One study explored AI-mediated language instruction in EFL classrooms and its effects on learning outcomes, motivation, and autonomous learning. Most studies have confirmed the positive influence on the enhancement of students’ learning proficiency and utilization of SRL strategies. Five studies adopted Zimmerman (2000) theoretical perspective on SRL, focusing on cognitive and metacognitive strategies. One of the studies demonstrated that technology-assisted self-regulated learning directly affects students’ enjoyment and self-efficacy.

Besides, 26% (nine studies) explored self-efficacy in the EFL context. Six studies discussed the role of SRL strategies on self-efficacy and the results indicated that SRL strategies can enhance students’ self-efficacy and subsequently influence learning performance. With in this group, one study’s participants are pre-service teachers, and the purpose of the study is to use the questionnaire to investigate the self-efficacy beliefs of these pre-service teachers when implementing SRL strategies in EFL classrooms.

There are four studies discussed the impact of the self-regulated strategy development (SRSD) instructional model. These studies explored diverse topics such as the impact on students’ writing performance, the influence on students’ reading comprehension skills, and the impact on students’ vocabulary learning. All these cases employed the five steps included in SRSD.

51% (18 studies) investigated the SBI and SRL in the English language learning contexts. These investigations covered a wide range of subjects, including scenario-based instruction, flipped classroom, explicit instruction, technology-assisted instruction, PBL, writing strategies and self-assessment and peer assessment. PBL is a systematic instructional method that engages students in inquiry through complex, authentic problems, and tasks, to facilitate their learning and acquisition of knowledge and skills (Markham et al., 2003). One study demonstrated the positive effects of self-assessment and peer assessment on the SRL, critical thinking, and problem-solving skills in EFL-speaking classes.

57% (20 studies) used intervention to assess the impact of SBI on students’ English language learning. Three studies employed mixed analysis of variances (ANOVAs) to analyze research data. Also, structural equation modeling (SEM) was used in three studies for data analysis. Among the 20 studies, 10 studies demonstrated the positive impact of SBI on writing skills. Through the implementation of SRL strategies, the intervention not only enhanced writing performance but also influenced the use of other strategies. One study indicated that SRSD had no effect on enhancing students’ learning abilities because the self-assessment of abilities before the intervention was inaccurate, resulting in no significant changes in students’ abilities. Although studies have all explored the positive effects of SBI on student learning outcomes and SRL strategies, some research has pointed out that due to the time limitations of intervention, the sustainable impact of SBI on SRL strategies cannot be established.

Building on these findings, the following chapter will turn to theoretical, practical, and methodological implications of the results. By synthesizing these insights, the discussion will offer actionable guidance for future research and practice, addressing identified gaps while advocating for more sustainable strategies in English language learning.

## 5 Discussion and implications

### 5.1 Theoretical implications

The selected studies investigate the self-regulated strategy development (SRSD) model, Zimmerman (2000) SRL theory, and Oxford (2016) Strategic Self-Regulation (S^2^R) model of language learning. The SRSD model (Harris and Graham, 1996) is applied specifically in writing instruction based on SRL strategies and has demonstrated the most significant influence among all strategies instruction approaches in the field of writing (Graham and Perin, 2007). Since 1985, many studies have applied the SRSD instructional model across various educational levels to improve students’ writing skills (Graham and Harris, 2009). Zimmerman (2000) SRL theory has led to extensive studies focusing on the relationship between motivational regulation strategies and cognitive, metacognitive, and social strategies, confirming that SRL is a multidimensional construct. In the process of SRL, motivational regulation strategies have been shown to influence other strategies (Zimmerman and Schunk, 2012). Additionally, studies on listening and writing tend to prefer Oxford (1990) taxonomy of language learning strategies. Researchers often choose Oxford’s model because of its comprehensive framework, which allows for an examination of both indirect and direct strategies employed in the language learning process. The multidimensional construct of SRL (Zimmerman, 2000) provides a foundation for researchers to further explore how different components (e.g., motivational regulation, cognitive strategies, and metacognitive strategies) interact and influence one another across various contexts. Building on this framework, future research could also consider analyze the long-term effects of motivational regulation strategies on students’ performance and persistence in language learning tasks.

### 5.2 Practical implications

Through the analysis of selected articles, research on the SBI and SRL strategies primarily targets students, teachers/instructors, curriculum designers, assessment designers, and teacher education/training program designers. The following sections will provide recommendations tailored to teachers, curriculum design, assessment, and teacher training.

To enhance students’ ability to use self-regulated learning (SRL) strategies, teachers can integrate strategy-based instruction (SBI) into their teaching practices. Teachers should recognize the impact of self-regulated learning strategies on students’ learning performance. Since SRL includes motivational regulation strategies, teachers should encourage students to consciously employ various motivational regulation strategies and enable them to engage in the learning process actively. Benson (2013) divided learning resources into two types: traditional learning resources, such as references and textbooks; and learning resources provided by modern educational technology, such as online platforms. Research on technology-assisted instruction has found that timely feedback can enhance students’ use of SRL strategies, thereby improving their learning performance. Therefore, teachers should prioritize providing feedback to students. Future studies can assess the role of emerging educational technologies and digital resources in facilitating SRL, identifying which tools most effectively support student engagement and strategy use. From the positive outcomes observed in studies, teachers require enough time for strategy instruction and should incorporate the teaching of strategies into teaching aims. Teachers should also consider that curriculum design needs to center on students rather than teachers, because teacher-centered may not fully reflect the effectiveness of SRL instruction. When designing the curriculum, curriculum designers should take into consideration the students’ rights to make judgments. Besides, differentiated instruction based on students’ various needs can significantly improve students’ SRL. For assessment, different kinds of assessments should be incorporated into the curriculum, especially self-assessment, and peer assessment. Peer assessment enables students to evaluate the learning of others to foster the development of student’s critical thinking skills. In addition, through self-assessment and peer assessment, teachers can better evaluate individual students, which leads to improved quality of teacher assessment. When providing feedback, teachers should pay attention to students’ overconfidence when encountering challenging tasks, and address instances where feedback indicates poor performance. In addition, it is suggested that teachers also need to assess students’ self-efficacy to prevent unrealistically high or low levels of self-efficacy from negatively impacting the learning behaviors of students. Regarding teacher training, teachers’ self-efficacy is positively related to the implementation of SRL strategies. If teachers lack self-efficacy, it will be a challenge for teachers to cultivate students’ awareness of SRL in the classroom. Therefore, teacher education programs need to provide teachers with knowledge of strategies, cultivate teachers’ awareness of strategy instruction, and establish requirements for strategy instruction. Future research can examine the effectiveness of professional development programs in equipping teachers with the knowledge and skills to implement strategies effectively in the classrooms.

### 5.3 Methodological implications

Analysis of the selected literature reveals that most studies employed intervention experiments and focused on small-sizes samples. In terms of educational background, most studies explored higher education level. Therefore, future research should expand to include broader language learning contexts and more diverse learner populations, such as region and educational levels (e.g., primary, secondary), to enhance the validity of the outcomes. Most studies have focused on the short-term effects of strategy instruction. To gain a more comprehensive understanding of the impact of strategy instruction on student learning, studies that offer a longitudinal perspective are required. Longitudinal research can also track the advancement of SRL strategies among learners, offering insights into how these skills evolve over time. Extended studies would facilitate an assessment of the sustainability of the effects of strategy instruction on student learning performance and SRL. Additionally, some studies employ questionnaires for data collection, however, due to the subjective of self-assessment, interviews and other forms of data collection (e.g., classroom observations) could be integrated for more comprehensive research. The research objects in the selected literature mainly focus on writing instruction. Future studies could explore the effects of SBI on other specific skills. Another area of research would be to consider individual differences, such as students’ motivation, personality style, and family background. Finally, the role of teachers in SBI instruction is crucial. However, there is limited research attention on the influence of teachers on students’ strategy instruction. Therefore, an important goal for further research is to investigate factors that influence teachers, such as self-efficacy and language proficiency.

## 6 Conclusion and limitations

The main goal of this systematic review was to examine research trends and shed light on research gaps of studies considering SBI and SRL in EFL/ESL contexts from 1994 to 2024. This study employed bibliometric analysis and content analysis. The bibliometric findings clearly indicated current research priorities through analysis of keywords, sources, and regions of selected papers. The content analysis explored the learning contexts (education level), research objects, intervention framework, practical implications, and suggestions for future research of the selected studies. The content analysis further examined the background and implementations of interventions to reveal the relationship between SBI and SRL strategy instruction. These characteristics encompass teaching contexts, duration, specific skills, and frameworks. The analysis suggests that future research could focus on examining the impact of SRL and SBI on specific language skills, such as reading, listening, grammar, and vocabulary, as well as exploring the conditions which these strategies have a more significant positive impact on students. Investigating the influence of AI-based instruction on student learning represents a promising direction for future studies. Additionally, the selection of learning context could expand beyond tertiary education level to include other educational levels, thereby broadening the applicability and practical significance of the findings.

A number of limitations need to be noted regarding the current study. Firstly, this study employed qualitative analysis to explore the selected empirical research, which indicates a lack of quantitative analysis. Further work might consider meta-analysis or other quantitative methods to obtain more comprehensive data and help researchers gain a more systematic evaluation of the implementations of SBI and SRL strategies in English language learning. Additionally, due to the relatively small number of eligible documents, the research findings have certain limitations. In order to obtain more comprehensive research data, this study only selected two databases. It is recommended that researchers apply both general databases and subject-specific databases, such as Google Scholar. While there are limitations, the outcomes of this study can provide suggestions for researchers intending to delve into the fields of SBI, SRL, and related areas. This is because the study examines existing research focuses, research trends, and research gaps.

## Data Availability

The original contributions presented in the study are included in the article, further inquiries can be directed to the corresponding author.
